# Organoids to Study Intestinal Nutrient Transport, Drug Uptake and Metabolism – Update to the Human Model and Expansion of Applications

**DOI:** 10.3389/fbioe.2020.577656

**Published:** 2020-09-11

**Authors:** Tamara Zietek, Pieter Giesbertz, Maren Ewers, Florian Reichart, Michael Weinmüller, Elisabeth Urbauer, Dirk Haller, Ihsan Ekin Demir, Güralp O. Ceyhan, Horst Kessler, Eva Rath

**Affiliations:** ^1^Chair of Nutritional Physiology, Technische Universität München, Munich, Germany; ^2^Pediatric Nutritional Medicine, Klinikum Rechts der Isar, Else Kröner-Fresenius-Zentrum für Ernährungsmedizin, Technische Universität München, Munich, Germany; ^3^Institute for Advanced Study, Department of Chemistry and Center for Integrated Protein Science (CIPSM), Technische Universität München, Garching, Germany; ^4^Chair of Nutrition and Immunology, Technische Universität München, Munich, Germany; ^5^ZIEL Institute for Food and Health, Technische Universität München, Munich, Germany; ^6^Department of Surgery, Klinikum Rechts der Isar, Technische Universität München, Munich, Germany; ^7^Department of General Surgery, HPB-Unit, School of Medicine, Acibadem Mehmet Ali Aydinlar University, Istanbul, Turkey; ^8^German Cancer Consortium (DKTK), Munich, Germany; ^9^CRC 1321 Modeling and Targeting Pancreatic Cancer, Klinikum rechts der Isar, School of Medicine, Technische Universität München, Munich, Germany

**Keywords:** peptidomimetics, acylcarnitine, glucose absorption, live cell imaging, fatty acid oxidation, PEPT1, competitive inhibition, 3R

## Abstract

Intestinal transport and sensing processes and their interconnection to metabolism are relevant to pathologies such as malabsorption syndromes, inflammatory diseases, obesity and type 2 diabetes. Constituting a highly selective barrier, intestinal epithelial cells absorb, metabolize, and release nutrients into the circulation, hence serving as gatekeeper of nutrient availability and metabolic health for the whole organism. Next to nutrient transport and sensing functions, intestinal transporters including peptide transporter 1 (PEPT1) are involved in the absorption of drugs and prodrugs, including certain inhibitors of angiotensin-converting enzyme, protease inhibitors, antivirals, and peptidomimetics like β-lactam antibiotics. Here, we verify the applicability of 3D organoids for *in vitro* investigation of intestinal biochemical processes related to transport and metabolism of nutrients and drugs. Establishing a variety of methodologies including illustration of transporter-mediated nutrient and drug uptake and metabolomics approaches, we highlight intestinal organoids as robust and reliable tool in this field of research. Currently used *in vitro* models to study intestinal nutrient absorption, drug transport and enterocyte metabolism, such as Caco-2 cells or rodent explant models are of limited value due to their cancer and non-human origin, respectively. Particularly species differences result in poorly correlative data and findings obtained in these models cannot be extrapolated reliably to humans, as indicated by high failure rates in drug development pipelines. In contrast, human intestinal organoids represent a superior model of the intestinal epithelium and might help to implement the 3Rs (Reduction, Refinement and Replacement) principle in basic science as well as the preclinical and regulatory setup.

## Introduction

Since 2009, when Sato et al. (2009) reported the generation and long-term *in vitro* cultivation of intestinal organoids, this technology had a tremendous impact on research on stem cell biology, basic medical science, disease modeling and personalized medicine. Previously, we reported on murine intestinal organoids for assessing nutrient transport and sensing as well as incretin hormone secretion (Zietek et al., 2015). Intestinal nutrient transporters are not only involved in the absorption of nutrients from ingested food, they also serve as sensors e.g., for glucagon-like peptide 1 (GLP-1) secretion and are able to transport certain drugs (Wenzel et al., 2002; Zietek and Daniel, 2015). Hence, intestinal transport processes and their interconnection to intestinal epithelial cell (IEC) metabolism and whole body metabolic state are relevant to a variety of diseases and represent potential therapeutic targets. Among these pathologies are intestinal diseases such as malabsorption syndromes and intestinal inflammation, as well as metabolic disorders including obesity and type 2 diabetes, but also pathologies treated with drugs that are actively absorbed and/or metabolized by enterocytes. For example, peptidomimetics like β-lactam antibiotics are substrates of peptide transporters (Wenzel et al., 2002), and a broad range of drugs is metabolized by intestinal cytochrome P450 enzymes (Xie et al., 2016). Furthermore, IEC not only constitute a barrier separating the host from its microbiota, epithelial metabolism serves as a gatekeeper of nutrient availability for the whole organism, and IEC fatty acid oxidation has been implicated in the control of eating (Langhans et al., 2011; Ramachandran et al., 2018). Yet, many aspects of nutrient absorption, drug bioavailability and enterocyte metabolism remain elusive, e.g., underlying causes of fructose malabsorption are still unknown (Ebert and Witt, 2016) and a model to predict the impact of chemical modifications of a drug on its oral bioavailability is missing (Ovadia et al., 2011; Rader et al., 2018b). Consequently, there is growing interest in model systems allowing to study intestinal nutrient absorption, drug transport and enterocyte metabolism.

Here, we verify the transferability of our previous uptake studies in murine intestinal organoids (Zietek et al., 2015) to human organoids, improved experimental protocols and expanded readouts for visualization of transport processes and metabolic analyses. Previously existing *in vitro* models such as Caco-2 cells, Madin-Darby canine kidney cell culture (MDCK) or rodent explant models (Ussing chamber, everted gut sac models) suffer from severe limitations, as they do not reflect human physiology due to their cancer origin (Pinto et al., 1983; Hidalgo et al., 1989) or their non-human origin, respectively. In contrast, human organoids closely reflect epithelial physiology in a region-specific resolution, conserve the phenotype of the donor and concurrently offer advantages of easy handling, long-term culture and expansion, as well as cryo-conservation (Almeqdadi et al., 2019). Since species-specific differences impede extrapolation of animal model-derived data to the human setup, focusing on human-based research models is essential for generating human-relevant data related to diseases and drug development. Currently, intestinal organoids are mostly discussed in the context of personalized medicine, allowing for individualized drug screening and prediction of drug responses in cancer patients and patients with cystic fibrosis (Kondo and Inoue, 2019; Phan et al., 2019; Schutgens and Clevers, 2020). However, organoids as superior model of the intestinal epithelium may additionally be used for basic studies on bioavailability of drugs, drug development, and toxicology testing, complementing and partly replacing animal testing (Grabinger et al., 2014; Takahashi, 2019). Our data support that already simple organoid culture protocols hold great potential in improving the toolbox of metabolic research and facilitating animal-free approaches.

## Materials and Methods

All relevant methods and materials can be found in the Supplementary Material.

## Results

### Culture Conditions Impact Organoid Cell Composition and Expression of Nutrient Transporters

**FIGURE 1:**
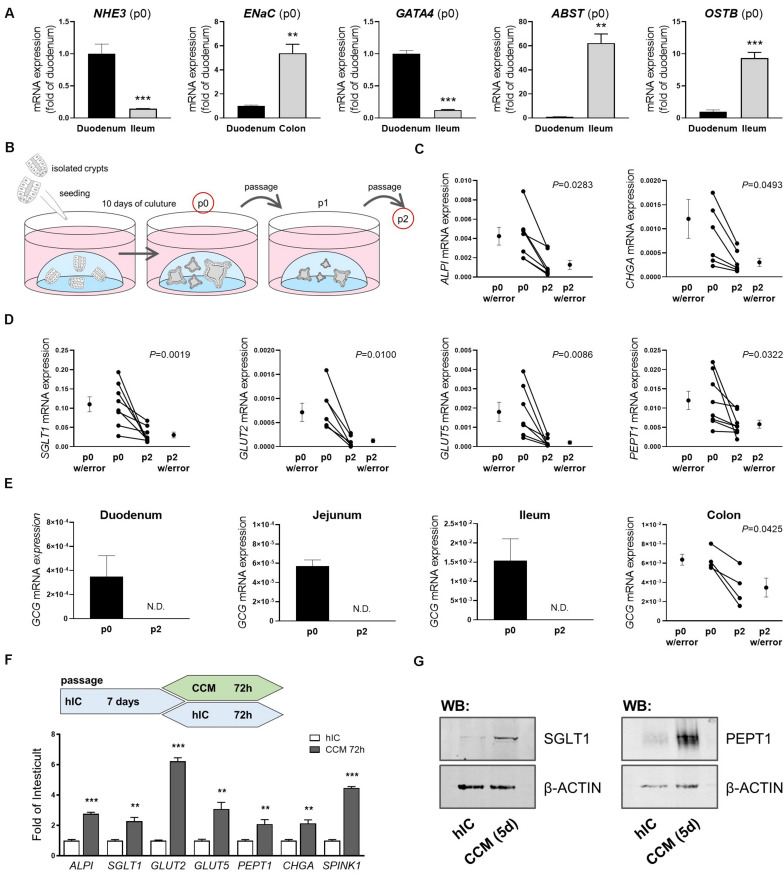
Culture conditions impact intestinal organoid cell composition and expression of nutrient transporters. **(A)** mRNA expression analyses of human organoids derived from different intestinal segments; site-specific genes are depicted (passage p0) **(B)** Schematic representation of the organoid culture from which samples were derived for analyses. **(C,D)** mRNA expression analyses of duodenal human organoids from passages p0 and p2. **(E)** mRNA expression levels of *GCG* in human organoids derived from different intestinal segments from passages p0 and p2. **(C–E)** Target gene expression normalized to *HPRT*. **(F)** Upper panel: schematic representation of the experimental setup from which samples were derived for mRNA expression analysis; lower panel: relative gene expression of CCM-cultured human duodenal organoids as fold of organoids cultured in Wnt-containing hIC medium. *HPRT* was used as housekeeper. Bars represent mean + SEM. **(G)** Protein expression of SGLT1 and PEPT1 in human organoids cultured in hIC and CCM medium for 5 days, respectively. β-ACTIN serves as loading control. **(A,F)** unpaired t tests (*n* = 5–6). **(C–E)** paired t tests (*n* = 5–6). Asterisks indicate significant differences *P < 0.05, **P < 0.01, ***P < 0.001; N.D. = Non-detectable; n.s. = non-significant; hIC = human IntestiCult medium, CCM = crypt culture medium.

Most nutrient and mineral uptake takes place in the small intestine, mediated by specific nutrient transporters located in the brush border membrane of enterocytes (Daniel and Zietek, 2015). A key property of intestinal organoids is that they are intrinsically programmed with their location-specific function and retain characteristics of their site of origin in culture (Middendorp et al., 2014). Consequently, differential expression of genes reported as site-specific (*GATA4*, *ABST*, *OSTB*) (Middendorp et al., 2014) as well as of *sodium-proton exchanger (NHE)3* and *epithelial sodium channel (ENaC)*, involved in epithelial transport processes in the small and large intestine, respectively, could be detected in human organoids derived from different intestinal segments (Figure 1A). The ratio of *NHE3* and *ABST* mRNA expression in duodenal- versus ileal-derived organoids reflected the ratio seen in the primary tissues used for crypt isolation, underlining the physiological relevance (Supplementary Figure 1A). In line, mRNA expression of the enterocyte marker intestinal alkaline phosphatase (*ALPI*) as well as the main apical glucose transporter sodium-glucose co-transporter (*SGLT*) 1, glucose transporter (*GLUT*) 2 mediating glucose and fructose fluxes at the basolateral membrane via facilitated diffusion, the apical fructose transporter GLUT5, and peptide transporter 1 (*PEPT1*) were observed in human organoids derived from the different regions (duodenum, jejunum, ileum) of the small intestine (Supplementary Figure 1B). *SGLT1* and *PEPT1* expression could also be detected in human colonic organoids (Supplementary Figure 1B) and chromogranin A (*CHGA*), a marker for enterodendocrine cells (EEC), was expressed in all intestinal segments investigated (Supplementary Figure 1B). Mature, differentiated enterocytes are a prerequisite to study transport processes, downstream signaling and metabolic responses. However, intestinal organoid culture media, including commercial ready-to-use media optimized for permanent propagation of human organoids contain Wnt factors and certain inhibitors that retain epithelial cells in an undifferentiated, stem cell-like state (Lindeboom et al., 2018). Accordingly, mRNA levels of the investigated genes significantly dropped after the first passage of organoids (Figures 1B–D). During extended culture (passages 4 to 8), expression remained stable at low levels (Supplementary Figure 1C). In particular, mRNA expression of *GCG*, encoding *i.a.* the incretin hormone glucagon-like peptide 1 (GLP-1) in EECs was rapidly lost. While completely undetectable in small intestinal organoids sampled after the second passage, expression levels were significantly diminished in colon-derived organoids (Figure 1E). Organoid differentiation can be steered into generation of distinct intestinal epithelial cell (IEC) subtypes like EECs (Petersen et al., 2015) or microfold (M) cells (de Lau et al., 2012) by addition of certain modulators like γ-secretase inhibitor or RANKL. *Vice versa*, withdrawal of compounds like Wnt3a or R-Spondin1 leads to general differentiation processes toward the enterocyte linage (Foulke-Abel et al., 2016; Lindeboom et al., 2018). In line, changing the growth medium of organoids from a commercially available medium suitable for long-term culture of human organoids (human IC (hIC), that contains non-available concentrations of growth factors and inhibitors) to the standard medium used for murine small intestinal organoid culture (crypt culture medium, CCM) containing epidermal growth factor (EGF), Noggin1, and R-Spondin1 but no Wnt factors, induced expression levels of *ALPI*, *SGLT1*, *GLUT2*, *GLUT5*, *PEPT1*, *CHGA* and *SPINK1* (Figure 1F). SPINK1 possesses structural similarities to EGF, and is associated with inflammatory states and various cancers, such as chronic pancreatitis (Hasan et al., 2018), inflammatory bowel disease and colon cancer (Ida et al., 2015). Western blot analysis of SGLT1 and PEPT1 protein expression confirmed the induction observed on mRNA levels (Figure 1G). A similar approach in murine small intestinal organoids, comparing CCM and murine IC (mIC) medium, yielded consistent results (Supplementary Figure 1D), highlighting the importance of appropriate culture conditions for functional readouts like transport assays or incretin hormone secretion in intestinal organoid cultures.

### Nutrient and Drug Transport in Human Intestinal Organoids

Simple sugars can be taken up by enterocytes via passive or active transport – and exit the enterocyte likewise. The mechanism of intestinal sugar absorption is still not fully understood, given that a variety of transporters of the sodium glucose co-transporter (SGLT) family and the family of facilitative glucose transporters (GLUT) with partly unknown specificities is involved (Thorens and Mueckler, 2010). Genetic variants of transporters contributing to intestinal sugar transport are associated with human diseases, such as glucose-galactose malabsorption and Fanconi-Bickel syndrome caused by mutations in SGLT1 (*SLC5A1*) and GLUT2 (*SLC2A2*), respectively (Martin et al., 1996; Santer et al., 1997). Particularly, fructose uptake gained increasing attention, as fructose consumption is rising over the last decades and is associated with developing cardiovascular diseases and type 2 diabetes (Johnson et al., 2007). Furthermore, the molecular basis of fructose malabsorption still remains elusive, but defective absorption is most likely. Hence, intestinal organoids which can be directly derived from patients and allow to picture the complex interaction of transporters might considerably advance science in this field.

**FIGURE 2:**
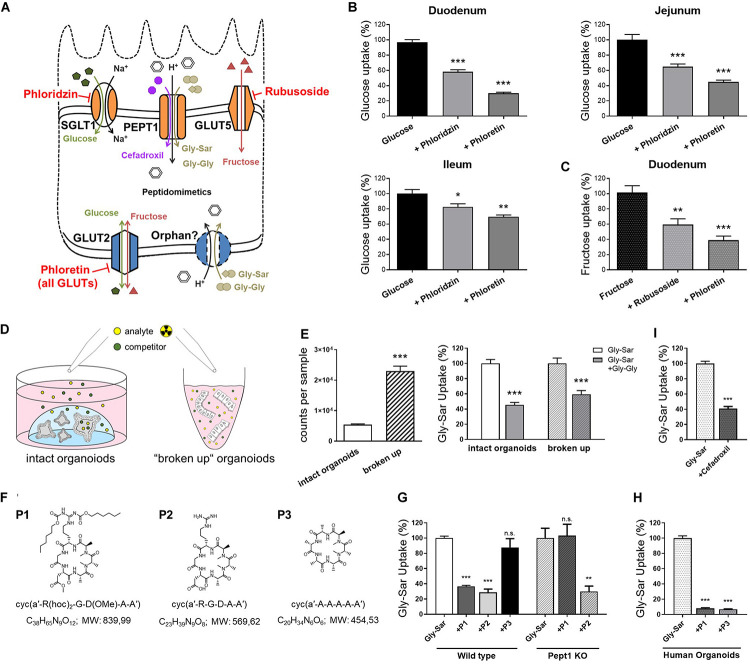
Nutrient and drug transport in human intestinal organoids. **(A)** Schematic illustration of the transporters investigated and inhibitors used. **(B)** Uptake of radiolabeled glucose in human organoids derived from different small intestinal segments. **(C)** Uptake of radiolabeled fructose in human duodenal organoids. **(D)** Schematic representation of the intact organoids- and “broken up” organoids approach for measuring transport activities. **(E)** Left: comparison of detected counts per sample for both approaches using the same amout of radiolabeled substrates and right: reduction of radiolabeled Gly-Sar uptake by the competitive inhibitor Gly-Gly depicted for both approaches. Reduction of uptake was not significantly altered comparing both approaches; One-way analysis of variance (ANOVA) followed by Tukey’s test. **(F)** Chemical structures and formulas of the peptidomimetics used. **(G)** Assessment of transport of peptidomimetics in a competition assay using radiolabeled Gly-Sar in murine small intestinal organoids derived from wild type and Pept1 knockout mice. **(H)** Similar approach to **(G)** using human duodenal organoids. **(I)** Reduction of radiolabeled Gly-Sar uptake using the antibiotic Cefadroxil as competitive inhibitor. **(B,C)** Unpaired t tests with Welch‘s correction. **(E,I)** Unpaired t tests. Bars represent mean + SEM. **(G,H)** One way ANOVA followed by Tukey’s test. For all experiments *n* = 5–6. Asterisks indicate significant differences *P < 0.05, **P < 0.01, ***P < 0.001.

Previously, we established a straightforward approach to assess nutrient and drug transport in murine intestinal organoids (Zietek et al., 2015). By using fluorescently (FITC) labeled dextrans, we were able to show that molecules of a size of 4 kDa rapidly reach the luminal compartment of murine organoids. Hence, radiolabeled substrates were simply added to the culture plates, keeping the organoids in their 3-dimensional environment (a dome of laminin-rich gel) (Zietek et al., 2015). As species-specific differences might result in misleading outcomes (Youhanna and Lauschke, 2020), we validated experimental procedures for human intestinal organoids, allowing for tackling human-specific research questions. After confirming translocation of 4 kDa FITC-dextrans also into the lumen of human organoids (Supplementary Figure 2A), we applied the experimental procedures established in murine intestinal organoids (Supplementary Figure 2B) to human organoids. First investigating uptake of glucose and fructose, we used different inhibitors for functional characterization of monosaccharide transport, the SGLT1 inhibitor phloridzin, the GLUT inhibitor phloretin and rubusoside, inhibiting fructose transport by GLUT5 (Figure 2A).

Glucose is a substrate for both, apical and basolateral GLUT transporters, with the electrogenic solute carrier SGLT1 as the main apical glucose transporter in the small intestine. GLUT5 represents an exception, transporting exclusively fructose at the apical membrane. Opposing, the uniporter GLUT2 mediates glucose and fructose fluxes at the basolateral membrane via facilitated diffusion, providing import as well as export capacities (Thorens and Mueckler, 2010; Roder et al., 2014; Figure 2A). Due to the experimental setup, substrates first reach the outside, i.e., basolateral side of the organoids and only subsequently, after reaching the organoid lumen via the paracellular route or by simple diffusion, the apical side. Therefore, it is not possible to target apical or basolateral transporters separately, yet the use of inhibitors enables to illustrate contributions of certain transporters. Thus, using glucose as substrate in combination with either phloridzin or phloretin in human organoids derived from different regions of the small intestine, resulted in the expected pattern of blunted glucose uptake, which was more pronounced with the pan-GLUT inhibitor phloretin as compared to the SGLT1 inhibitor phloridzin (Figure 2B). In line, fructose transport could be diminished by phloretin, and to a lesser extent, by the GLUT5-inhibitor rubusoside (Figure 2C) as well as glucose (Supplementary Figure 2C) in human duodenal organoids.

Aiming at improving the experimental protocols, a second approach for investigating transport processes was established, using non-enzymatically dissociated (“broken up”) organoids instead of intact organoids (Figure 2D). In this case, human duodenal organoids were exposed to the radiolabeled dipeptide glycyl-sarcosin (Gly-Sar), a hydrolysis-resistant model substrate of the peptide transporter PEPT1. Peptide transport over the plasma membrane occurs in cotransport with protons and allows transport of di- and tripeptides against a substrate gradient[24]. Additionally, PEPT1 also facilitates absorption of drugs and prodrugs, including certain inhibitors of angiotensin−converting enzyme (ACE), protease inhibitors, antivirals and peptidomimetics such as aminocephalosporins (Ganapathy et al., 1995; Shu et al., 2001; Sugano et al., 2010; Kottra et al., 2013). Next to PEPT1-mediated substrate fluxes at the apical membrane, a not yet genetically identified system for basolateral peptide uptake with similar features to PEPT1 has been described (Berthelsen et al., 2013). Although radiolabeled transport assays are very sensitive, costs of labeled substrates are a major drawback. Hence, reducing the amount of substrates needed for experiments is desirable. Comparing intact organoids and “broken up” organoids exposed to the same concentrations of radiolabeled Gly-Sar, a 4-fold increase in signal intensity was observed. Competitively inhibiting Gly-Sar uptake by the dipeptide glycyl-glycine (Gly-Gly) demonstrated a significant reduction of Gly-Sar uptake in both approaches, with the extent of reduction not being different between intact and “broken up” organoids (Figure 2E). Consequently, using “broken up” organoids instead of intact organoids represents a possibility not only to reduce costs but also to target basolateral and apical transporters at the same time.

As mentioned before, peptide transporters also play a role in drug uptake, including peptidomimetics. Peptidomimetics are compounds mimicking a peptide or protein, which possess the ability to interact with a biological target to exert agonistic or antagonistic effects (Giannis and Kolter, 1993; Marshall and Ballante, 2017). Hence, they have a great potential in drug discovery, exerting drug-like properties (Rader et al., 2018a). For example, peptidomimetics have been designed for cancer therapy, e.g., to induce apoptosis (Walensky et al., 2004), sensitize cancer cells to chemotherapeutics (Greer et al., 2011), or specifically targeting integrins for interfering with angiogenesis and other aspects of tumor biology (Mas-Moruno et al., 2010; Nieberler et al., 2017). Primary goals in the development of orally available peptides are improving their intestinal transport and enhancing their stability to enzymatic degradation. Common strategies comprise the use of cyclic peptides, as well as D- instead of L-amino acids and *N*-methylation to increase metabolic stability (Rader et al., 2018a). For example, Cilengitide, a cyclic pentapeptide with one D-amino acid and one *N*-methylation is completely stable in humans and is excreted with a half-life of 4 h without any metabolization (Becker et al., 2015). Yet, intestinal permeation from the lumen into the bloodstream remains a major challenge. Structural changes affect intestinal and cellular permeability, and a change in one methyl position already can greatly impact permeability properties (Ovadia et al., 2011). Oral availability (crossing the gastrointestinal wall to reach the circulation) can be mediated via paracellular or transcellular mechanisms, including active transporters (Rader et al., 2018a). To date, it is not possible to predict the impact of certain chemical modifications on the transport/oral bioavailability of drug candidates (Rader et al., 2018b), therefore screening systems are required. Common tools to evaluate permeability properties of peptide drugs include Caco-2 monolayers and the side-by-side diffusion chamber (Ussing chamber), however, both systems are poorly correlative (Jezyk et al., 1999; Bermejo et al., 2004; Ovadia et al., 2011) and face major disadvantages. Caco-2 cells, even though known to possess a rather small intestinal phenotype (Yee, 1997), were originally derived from a colon carcinoma, and phenotypic as well as functional characteristics highly differ from native human enterocytes (Harwood et al., 2016). For example, Caco-2 cells exhibit tighter junctions compared to the small intestine of human (Matsson et al., 2005) and were found not to be appropriate for evaluating active, carrier-mediated peptide drug absorption (Jezyk et al., 1999). In contrast, Ussing chamber approaches, mainly using excised rat tissue better reflect physiology but suffer from potential species differences and large numbers of animals needed for screening. Hence, we tested the applicability of intestinal organoids as a new tool to evaluate the absorption properties of peptidomimetics. Three different cyclic hexapeptides **(P1, P2, P3)** (Figure 2F) were tested that were originally developed via a stepwise library approach: First a library of more than 55 different *N*-methylated alanine peptides of the general structure cyclo(D-Ala-L-Ala_5_) were synthesized and investigated in a Caco-2 assay (Ovadia et al., 2011). Peptides identified as highly permeable (including **P3**) were subsequently functionalized by substitution of neutral Ala residues with the integrin-binding tripeptide sequence RGD. Among them, one compound (**P2**), has been identified with similar high activity and selectivity as Cilengitide (sub-nanomolar affinity for integrin αvβ3, high selectivity against other integrins) (Weinmuller et al., 2017). However, **P2** lacked permeability due to charges in the cyclic *N*-methylated alanine-peptides. To overcome this limitation, charged residues were protected with lipophilic protecting moieties (two hexyloxycarbonyl (Hoc) groups and conversion of the carboxylic side chain of Asp into a neutrally charged methyl ester). The resulting compound **P1** showed both, permeability in the Caco-2 assay and biological activity after oral administration in mice (Weinmuller et al., 2017). To test the involvement of active peptide transporter-mediated uptake in the permeability properties of **P1-P3**, we evaluated the ability of the three cyclic hexapeptides to competitively inhibit the uptake of radiolabeled Gly-Sar in murine small intestinal organoids derived from wild type and Pept1-deficient mice. In this assay, we identified **P1** as a potential substrate for active transport mediated by Pept1, **P2** to be actively transported independently of Pept1, and in contrast, **P3** showed no signs of peptide transporter-mediated uptake in murine organoids (Figure 2G). Subsequently testing **P1** and **P3** in human duodenal organoids, both peptides were able to significantly reduce radiolabeled Gly-Sar uptake, indicating **P1** and **P3** to be substrates for peptide transporter-mediated uptake in humans (Figure 2H). These data highlight the suitability of intestinal organoids to screen for transporter-mediated uptake of drug candidates, a process that might have been underappreciated in Caco-2 assays due to lack of physiological transporter expression, but contributes to oral availability. Additionally, efflux processes that limit drug absorption might be evaluated in detail in organoid systems (Schumacher-Klinger et al., 2018). Concomitantly, these data point toward potential species-specific transport phenotypes as already described for PEPT1 (Kottra et al., 2013). Accordingly, we could also confirm transport of the peptide-like β-lactam antibiotic cefadroxil, that has been previously described as PEPT1 substrate (Ganapathy et al., 1995; Zietek et al., 2015), in human duodenal organoids (Figure 2I).

In conclusion, these results underline the superior properties of human intestinal organoids for studying nutrient and drug uptake. Since organoids retain location-specific properties of their site of origin, absorption could even be determined at an intestinal region-specific resolution.

### Visualization of Intestinal Peptide Transport Processes

**FIGURE 3:**
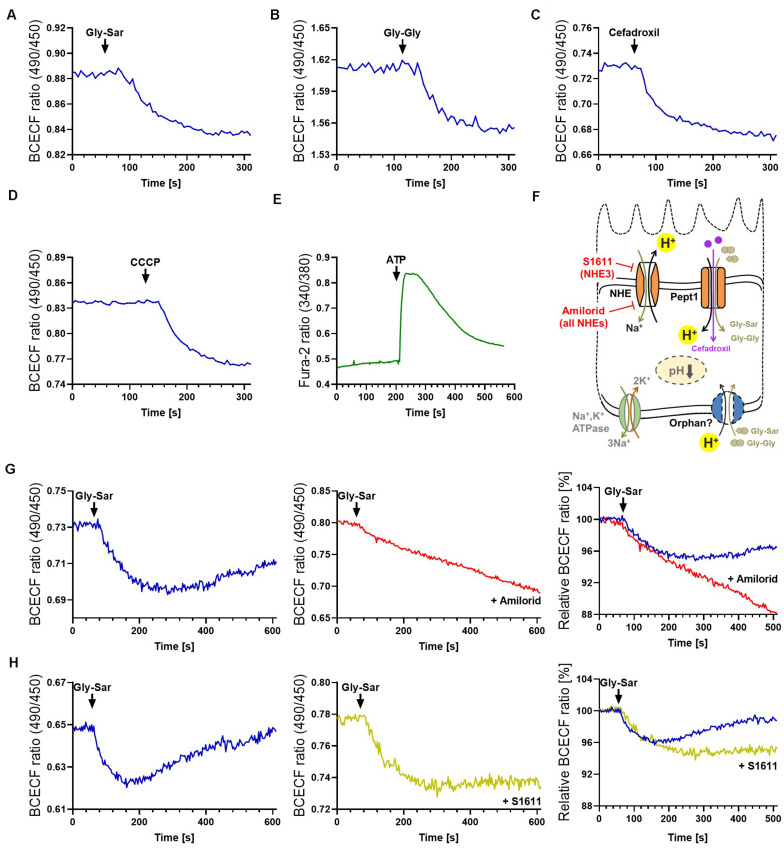
Visualization of intestinal peptide transport processes. Intracellular acidification visualized by BCECF-AM induced by transport of peptide-transporter substrates **(A)** Gly-Sar and **(B)** Gly-Gly, **(C)** by the antibiotic Cefadroxil and **(D)** the protonophore CCCP. **(E)** Calcium responses to ATP stimulation visualized by Fura-2. Intracellular acidification induced by the antibiotic Cefadroxil. **(F)** Schematic illustration of the transporters investigated and inhibitors used. **(G)** Course of intracellular acidification induced by Gly-Sar exposure for an extended time frame (left) with and (middle) without the NHE-inhibitor Amilorid; right: overlay of both curves giving relative BCECF ratios. **(H)** Similar approach to **(G)** using the NHE3-inhibitor S1611. **(A–E)** human duodenal organoids, **(G,H)** murine small intestinal organoids. For data analysis, whole organoids were selected and no background correction was applied. Analyses were performed on several organoids derived from independent cultures and representative measurements are shown.

It has been reported that fluorophore-conjugated dipeptides with a high-affinity for PEPT1 were able to block transport of Gly-Sar, however, they failed to be transported (Abe et al., 1999; Kottra et al., 2013). To exclude similar effects, either specific inhibitors can be applied (for example Lys-z-NO2-Val, a specific PEPT1-inhibitor) or downstream effects of transport processes can be investigated. Thus, we extended our previously established protocol for visualization of intracellular signaling by life-cell imaging of murine intestinal organoids (Zietek et al., 2015), to human organoids and drug transport-induced signaling events. As mentioned before, peptide transport over the plasma membrane occurs in cotransport with protons, leading to cytosolic acidification of enterocytes (Chen et al., 2010). Hence, intracellular changes immediately reflect transport activities and provide direct evidence for substrate fluxes. A drop in pH can be visualized by live-cell imaging using fluorescent probes (Chen et al., 2010; Zietek et al., 2015). Employing the pH-indicator BCECF-AM, intracellular acidification was demonstrated in human duodenal organoids upon exposure to Gly-Sar, Gly-Gly as well as cefadroxil and the carbonyl cyanide m-chlorophenyl hydrazine (CCCP, an ionophore used as a positive control) (Figures 3A–D). Stimulating organoids with CCCP subsequent to administration of Gly-Sar, Gly-Gly, and cefadroxil caused an additional decline in intracellular pH, indicating the physiological range of observed responses (Supplementary Figure 3A). As expected, neither glucose nor fructose (used as negative controls) led to an intracellular acidification of enterocytes (Supplementary Figure 3B). Since live-cell imaging of calcium fluxes is routinely applied in pharmacological screenings to detect activation of receptors by a putative ligand/drug, and many transporter activities (e.g., PEPT1) (Wenzel et al., 2002) and intracellular translocation events (e.g., GLUT2) (Kellett et al., 2008) are regulated via intracellular calcium, we confirmed the applicability of the calcium-indicator Fura-2-AM in human organoids. In accordance to literature, robust signals were obtained upon ATP-mediated increases in intracellular calcium (Figure 3E). For both dyes, BCECF-AM and Fura-2-AM, excellent dye-loading efficiency was observed (Supplementary Figure 3C).

For continuous peptide uptake, IECs need to maintain the transmembrane ionic gradients and furthermore, augmented or prolonged acidification of the cell by proton symport of peptide transporters has to be avoided. Hence, protons are exported in exchange with Na^+^ by sodium−proton exchangers (NHEs) (Figure 3F, Supplementary Figure 1A). In enterocytes, several types of NHEs are expressed, and NHE3 specifically has been shown to be required for proper PEPT1-mediated transport (Chen et al., 2010). Importantly, NHE-function is targeted by both, clinically relevant drugs as well as bacterial toxins. The distinct role of NHE3 in Na^+^ absorption during normal digestion and in acute and chronic diarrheal diseases has been explored in human enteroids by Foulke-Abel et al. (2016), underlining the possibility to identify drug targets in this system. To illustrate the function of NHEs in general and NHE3 in particular in the context of active peptide transport in organoids, we used two different inhibitors: Amiloride, an FDA-approved inhibitor of NHEs, and S1611, which predominantly acts on NHE3 (Wiemann et al., 1999). As expected, both inhibitors prevented the recovery of intracellular pH to basal levels as observed in non-treated murine organoids following exposure to Gly-Sar (Figures 3G,H left). In accordance to their specific inhibitory spectrum, amiloride led to a continuous influx of protons in the observed time span (Figure 3G), while S1611 treatment resulted in a stable intracellular pH level below base line (Figure 3H). To decipher biology and functional characteristics of intestinal transporters it is very important not only to quantify transport of substrates, but also to take intracellular downstream effects and signaling into account, as presented above. These data highlight the high-resolution measurements possible in intestinal organoids.

### Metabolite Analysis in Intestinal Organoids

Metabolism in IECs has gained increasing attention, not only due to the expression of key drug metabolizing enzymes, including cytochrome P450 3A4 (CYP3A4), in small intestinal epithelial cells, that are prone to diet-drug interactions (Lown et al., 1997). IEC and whole body metabolism are tightly interrelated via production of incretine hormones (Zietek and Rath, 2016) and factors like Fgf15 (Kliewer and Mangelsdorf, 2015) by enteroendocrine cells and enterocytes, respectively, and vice versa, IECs are targets of remote-tissue metabolic signals such as insulin and leptin signaling (Yilmaz et al., 2012; Le Drean and Segain, 2014). In the gastrointestinal tract, carbohydrates, peptides and lipids are broken down and absorbed by enterocytes. Subsequently, they serve as substrates for cellular energy generation or for interconversions and distribution to the whole organism via transfer into the circulation. Hence, IEC metabolism also profoundly impacts availability and quality of nutrients, constituting an initial check point between diet and host. In this context, the intestinal microbiota plays an additional key role, as a source of bacterial metabolites such as short chain fatty acids (SCFAs) including butyrate. IEC metabolism and exposure to certain nutrients furthermore relates to diseases, for example high-fat diets were shown to enhance tumorigenicity of intestinal progenitors (Beyaz et al., 2016) and SCFAs and lactate promote intestinal healing processes (Lee et al., 2018; Parada Venegas et al., 2019). Despite the fact that general metabolic functions of enterocytes are understood, many open questions remain, including whether the small intestine can act as a site for gluconeogenesis, which seems to be species-dependent (Sinha et al., 2017; Potts et al., 2018; Varga et al., 2019) or how carbohydrate and lipid absorption and metabolism interact. Metabolomic approaches are key technologies allowing to tackle such questions by enabling analysis of metabolic events in a large scale and high throughput manner.

**FIGURE 4:**
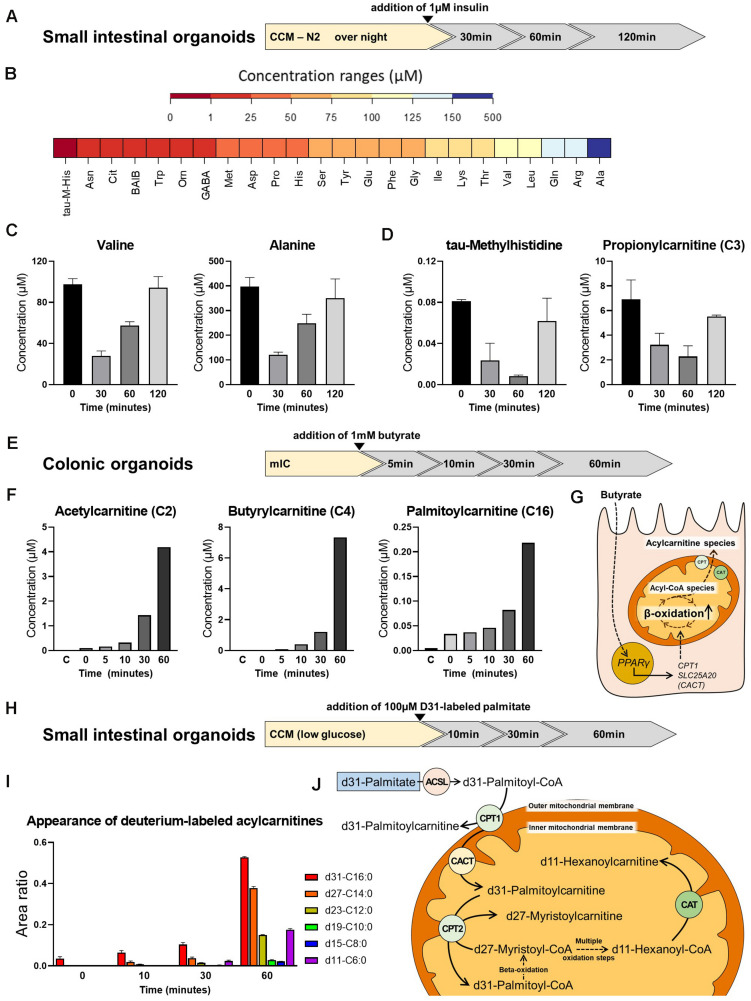
Metabolite analysis in intestinal organoids. **(A)** Schematic representation of the experimental setup from which samples were derived for analyses shown in panel **(B,C)**. **(B)** Range of amino acid (AA) concentrations detected in organoids. **(C)** Concentration of valine and alanine at different time points after insulin stimulation. **(D)** Concentration of tau-methylhistidine, a marker compound for proteolysis, and propionylcarnitine (C3), a typical intermediate in the breakdown of valine, at different time points after insulin stimulation. **(E)** Schematic representation of the experimental setup from which samples were derived for analyses shown in panel **(F)**. **(F)** Concentration of the acylcarnitine species Acetylcarnitine (C2), Butyrylcarnitine (C4), and Palmitoylcarnitine (C16) at different time points after addition of butyrate. **(G)** Proposed mode of action for the effect of butyrate on beta-oxidation. **(H)** Schematic representation of the experimental setup from which samples were derived for analyses shown in panel **(I)**. **(I)** Appearance of deuterium-labeled acylcarnitines at different time points after addition of deuterium-labeled d31-palmitate. **(J)** Schematic illustration of carnitine acyltransferases involved in the generation of the acylcarnitine species detected. **(B,C,F,I)** Representative results from three independent organoid cultures. ASCL, long-chain acyl-CoA synthetase; CPT, carnitine palmitoyltransferase; CAT, carnitine acetyltransferase; CACT, carnitine-acylcarnitine translocase.

To test the feasibility of metabolic measurements in intestinal organoids, we applied different experimental schemes to replicate/validate effects described in literature. First, we determined the effect of insulin on amino acid (AA) and acylcarnitine levels in small intestinal organoids. Murine intestinal organoids were deprived of insulin-containing N2 medium supplement (yet, the B27 supplement contains residual insulin in a n/a concentration) over night, stimulated with 1 μM insulin and AAs and acylcarnitines were measured after 0, 30, 60, and 120 min (Figure 4A). All proteinogenic amino acids could be detected in small intestinal organoids at concentration ranges given in Figure 4B. Insulin is known to promote anabolism, affecting both, processes of protein synthesis and proteolysis. Enterocytes respond to insulin signals and develop insulin resistance under conditions of obesity-related inflammation (Monteiro-Sepulveda et al., 2015), and in particular, it was demonstrated that insulin deprivation reduces small intestinal protein biosynthesis, an effect that could be rescued by insulin treatment (Charlton et al., 2000). In line, concentrations of valine and alanine responded fast to insulin stimulation showing maximal reduction 30 min after addition of insulin (Figure 4C), consistently with most other AAs (data not shown), indicating a shift in protein turnover toward an enhanced net incorporation of AAs in proteins. In parallel, tau-methylhistidine, a marker compound for proteolysis and propionylcarnitine (C3), a typical intermediate in the breakdown of valine, isoleucine, methionine and threonine were diminished with lowest levels observed 60 min after insulin stimulation (Figure 4D), confirming also the inhibitory effect of insulin on proteolysis in intestinal organoids.

Next, we depict the effect of butyrate on acylcarnitine profiles in murine large intestinal organoids. In this approach, 1mM butyrate was added and shifts in acylcarnitines were measured 0, 5, 10, 30, and 60 min afterward (Figure 4E). Butyrate has been shown to broadly affect colonocyte metabolism, including glucose utilization (Donohoe et al., 2012) and fat oxidation (den Besten et al., 2015), in turn regulating cell cycle progression and proliferation (Donohoe et al., 2012). In accordance to literature, a clear effect of butyrate on saturated acylcarnitines, comprising short-, medium- and long-chain acylcarnitines was observed, with acetylcarnitine, butyrylcarnitine and palmitoylcarnitine increasing to maximal concentrations 60 min after butyrate addition (Figure 4F). A proposed mechanism explaining the effect of butyrate involves the butyrate transporter SLC5A8 and the butyrate receptor GPR109A expressed by coloncytes (Cresci et al., 2010), mediating activation of PPARγ signaling, in turn increasing expression of carnitine palmitoyl-CoA transferase (CPT1) and carnitine-acylcarnitine translocase (SLC25A20/CACT) to enhance mitochondrial beta-oxidation (Vanhoutvin et al., 2009; den Besten et al., 2015; Figure 4G).

Last but not least, we followed the breakdown of d31-labeled palmitic acid, in which all 31 hydrogen atoms are replaced by deuterium atoms, in small intestinal organoids. Stable isotope labeling enables following the fate of the labeled fatty acid within the enterocyte, being either subjected to chain-shortening during beta-oxidation and conversion to the respective acylcarnitine species for energy generation, or being reesterified, and incorporated into chylomicrons for systemic supply. Importantly, sensing dietary fat via fatty acid oxidation in enterocytes has been implicated in the control of eating (Langhans et al., 2011), and modulation of enterocyte metabolism might affect whole body glucose homeostasis and the development of diet-induced obesity (Schober et al., 2013; Ramachandran et al., 2018). Prior to addition of d31-labeled palmitic acid, murine small intestinal organoids were incubated with CCM prepared with low-glucose DMEM/F12 for 24 h. Low-glucose DMEM/F12 contains 1g/L glucose, corresponding to 5.5 mM glucose, which is within the physiological range. Appearance of deuterium-labeled acylcarnitines were determined 0, 10, 30, and 60 min after addition of d31-palmitic acid (Figure 4H). Indicating beta-oxidation, we could detect chain-shortened, deuterium-labeled acylcarnitine species (Figure 4I). The conversion of the long-chain fatty acids to their acylcarnitine species is known to be mediated by carnitine palmitoyltransferase 1 and 2 (CPT1 and CPT2), while short-chain acylcarnitine species are formed by carnitine acetyltransferase (CAT) (Figure 4J). Carnitine octanoyltransferase (COT) located in peroxisomes is responsible for the conversion of medium-chain fatty acids (Violante et al., 2013). Contrarily, CPT1 is located in the outer mitochondrial membrane and thus may convert the added d31-palmitic acid directly to d31-palmitoylcarnitine (Bonnefont et al., 2004). In line, immediately after addition of d31-palmitic acid (*t* = 0), a peak of d31-palmitoylcarnitine (d31-C16:0) was detected, that increased in subsequent time points (Figure 4I). Shorter fatty acid intermediates are formed within the mitochondria and their respective acylcarnitine species are generated by CPT2 and CAT, located in the inner mitochondrial membrane. Consistent with the sequential removal of 2-carbon units during beta-oxidation, d27-myristoylcarnitine (d27-C14:0) and to a lesser extent d23-dodecanoylcarnitine (d23-C12:0) could already be seen after 10 min of incubation, whereas d19-decanoyl-, d15-octanoyl- and d11-hexanoylcarnitine appeared 30 min after addition of d31-palmitic acid. All of these intermediates showed increasing peaks for *t* = 60 (Figure 4I). Of note, the larger peaks of d11-C6, as compared to d19-C10 and d15-C8 after 30 and 60 min might be explained by a higher preference of CAT for short-chain fatty acid substrates (C2 to C6).

In summary, intestinal organoids are an excellent model system close to physiology to explore cellular metabolism and the applied metabolic readouts could be adapted easily to the 3D culture. Human organoids, constituting the most relevant model, are superior to animal (rodent)-derived organoids and (cancer) cell lines, especially in the context of metabolism and diseases, since metabolic properties differ between species and alterations in the cellular metabolism are part of many pathologies. Thus, human organoids hold great potential to answer remaining questions on intestinal metabolism and to identify drug targets to improve overall metabolic health.

## Conclusion

Taken together, our results demonstrate that intestinal organoids cultured in 3D, embedded in a laminin-rich gel dome, the most basic and probably least cost and labor extensive culture protocol, is suitable for a broad range of measurements in the field of intestinal transport and metabolic studies. Beyond these applications, many other readouts are possible in this setup, for example assessment of proteasome activity (Supplementary Figure 2D), which is of interest in the context of proteasome inhibitors, an important class of drugs in the treatment of different types of cancer (Fricker, 2020).

Simple improvements and “tricks” like changing the medium composition to promote differentiation or to “break up” organoids prior to uptake studies help further enhancing results and reducing costs. Implementing other culture protocols like organoids with reversed polarity (in which the apical side faces outward) (Co et al., 2019) or organoids seeded in a 2D layer in transwell plates (VanDussen et al., 2015; Wang et al., 2019) are additional roads to go. Paracellular transport of fluorescein, transcellular transport of propranolol, and basolateral efflux of rhodamin123, a substrate of p-glycoprotein (MDR1) have been measured in a model in which human organoid-derived cells are seeded as a 2D monolayer on a porcine small intestinal scaffold (Schweinlin et al., 2016), complementing our animal-free approach focusing on active, transporter-mediated substrate uptake.

The field of applications for organoids is still rapidly growing, and there is a trend toward more complex and sophisticated organoid-based model systems. For example, co-cultures with bacterial and viral pathogens and immune cells (Yin et al., 2015; Dutta and Clevers, 2017), as well as approaches to reproduce the complex tissue environment comprising continuously flowing fluid systems, or to reflect multi-organ interactions (organoids on a chip), have been developed (Almeqdadi et al., 2019). These systems provide a microenvironment to study the impact of oxygenation, mechanical stress, and tissue communication via soluble factors and will further advance intestinal research. Yet, to date they remain very expensive tools in highly specialized laboratories not suitable for broad applications (Almeqdadi et al., 2019). In contrast, the intestinal organoid culture protocols and methods presented here represent *in vitro* models that already now allow for partly replacement and reduction of animal numbers needed for research and testing.

Although the methodologies that we have established are applicable to mouse and human organoids, the human organoid technology should be focused when targeting human-related issues. Drug development success rates are particularly low in widespread diseases such as diabetes (Ali et al., 2018) or cancer (Mak et al., 2014). Only 5 to 10 percent of drugs proven as safe and effective in preclinical animal studies make it to the market (Arrowsmith, 2012; Thomas et al., 2016). Species-specific differences and hence poor transferability from animal models to humans is the main reason for this high failure rate (Arrowsmith and Miller, 2013; Cook et al., 2014; Mullard, 2016).

In light of this, we provide innovative approaches for physiologically relevant *in vitro* testing in the field of intestinal research and metabolomics. In particular, the use of human organoids in this context is a highly valuable tool for drug discovery and testing as well as for human-relevant disease modeling.

## Data Availability Statement

All datasets presented in this study are included in the article/Supplementary Material.

## Ethics Statement

The studies involving human participants were reviewed and approved by the Ethics Committee of the Medical Faculty of TUM. The patients/participants provided their written informed consent to participate in this study. The animal study was reviewed and approved by the Committee on Animal Health and Care of the local government body of the state of Upper Bavaria (Regierung von Oberbayern).

## Author Contributions

TZ contributed to study conception and design, human organoid culture, data acquisition, analysis and interpretation, and drafting and revising the article. PG contributed to data acquisition, analysis and interpretation, and drafting and revising the article. ME contributed to data acquisition, organoid culture, and analysis and interpretation. FR and MW contributed to synthesis of peptidomimetics. EU contributed to organoid culture and analysis of protein expression. DH contributed to critically revising the article. ID and GC provided material for organoid preparation. HK contributed to study conception and critically revising the article. ER contributed to study conception and design, murine and human organoid culture, data acquisition, analysis and interpretation, and drafting and revising the article. All authors contributed to the article and approved the submitted version.

## Conflict of Interest

The authors declare that the research was conducted in the absence of any commercial or financial relationships that could be construed as a potential conflict of interest.
