# Supplementary material for: Organoids to Study Intestinal Nutrient Transport, Drug Uptake and Metabolism – Update to the Human Model and Expansion of Applications
Source: Front Bioeng Biotechnol. 2020 Sep 11;8:577656. doi: 10.3389/fbioe.2020.577656 (PMC7516017; doi:10.3389/fbioe.2020.577656)
Supplement: Supplementary file 1 [file Data_Sheet_1.pdf]

Organoids to Study Intestinal Nutrient Transport, Drug Uptake and Metabolism – Alternatives to Animal Testing

Supplementary Material

Table of contents

Table of contents 1

1. Methods and Materials 2
1.1. *Ethics statement* 2
1.2. *Primary crypt isolation and intestinal organoid culture* 2
1.3. *RNA isolation, reverse transcription and quantitative real-time PCR* 2
1.4. *Western blot analysis* 3
1.5. *Permeability measurement* 3
1.6. *Glucose and Fructose transport studies* 3
1.7. *Peptide and drug transport studies* 4
1.8. *Imaging of intracellular acidification* 4
1.9. *Imaging of intracellular calcium concentrations* 5
1.10. *Quantification of amino acid and acylcarnitine concentrations using targeted LC-MS/MS* 5
1.11. *Proteasome activity measurement* 6
1.12. *Statistical analysis* 6

2. Supplementary Table
Table 1: *Primer sequences for quantitative real-time PCR* 7

3. Supplementary Figures
Sup. Figure 1: *Culture conditions impact organoid cell composition and expression of nutrient transporters* 8
Sup. Figure 2: *Nutrient and drug transport in human intestinal organoids* 9
Sup. Figure 3: *Visualization of intestinal peptide transport processes* 10

4. References 11

1. Methods and Materials

1.1. Ethics statement

For generation of human organoids, the use of surgically resected human tissue samples was approved by the Ethics Committee of the Medical Faculty of TUM and all participants in this study gave their written informed consent. The maintenance and breeding of mouse lines for organoid generation was approved by the Committee on Animal Health and Care of the local government body of the state of Upper Bavaria (Regierung von Oberbayern) and performed in strict compliance with the directive 2010/63/EU of the European Parliament on the protection of animals used for scientific purposes.

1.2. Primary crypt isolation and intestinal organoid culture

Human intestinal organoids were derived from macroscopically healthy tissue of surgical specimen from the small and large intestine (duodenum, jejunum, ileum, and colon, respectively). Tissue was prepared as previously described [1] and digested using Gentle Cell Dissociation Reagent (GCDR, STEMCELL Technologies Grenoble, France) according to the manufacturer´s instructions. 500 crypts were embedded in 30µl matrigel (BD Biosciences, Franklin Lakes, NJ) per well, resulting in approx. 100 mature organoids, and cultured in 48 well plates. Human organoids were cultivated in Human Intesticult medium (STEMCELL Technologies, Grenoble, France) (hIC) containing Wnt factors or, whenever indicated in CCM (see murine intestinal organoid culture). Every 10 days, human organoids were passaged and re-seeded in fresh matrigel.
All murine organoids were obtained from an internal biobank, thawed and taken into culture for experiments. Initially, mice used for intestinal crypt isolation (Wild type (Wt) C57BL/6 mice and Pept1$^{-/-}$ mice (C57BL/6 background) [2]) were 8–18 weeks of age. Briefly, after sacrificing mice by CO2 inhalation, the small intestine was removed and trimmed free of adjacent tissues. Primary intestinal crypts from the proximal 10 cm of the small intestine were isolated by tissue incubation in 2mM EDTA (Fisher, Dreieich, Germany) and cultured as described elsewhere [1]. Crypts were embedded in 25µl matrigel (BD Biosciences, Franklin Lakes, NJ) and cultured in 48 well plates. Murine organoids were cultivated in crypt culture medium (CCM) consisting of advanced DMEM/F12 medium (Gibbco, Cincinnati, OH) containing 2mM GlutaMax (Gibbco), 10mM HEPES, penicillin, streptomycin and amphotericin (all Sigma-Aldrich, Taufkirchen, Germany) supplemented with N2, B27 (both Gibbco), 1mM N-acetylcystein (Sigma- Aldrich), 50ng/mL EGF (ImmunoTools, Friesoythe, Germany), 100ng/mL noggin and 0.5µg/mL R-spondin 1 (both PeproTec, Rocky Hill, NJ) or whenever indicated in Murine Intesticult medium (STEMCELL Technologies, Grenoble, France) (mIC) containing Wnt factors. Murine Organoids were passaged and re-seeded in fresh matrigel every 7 days of culture.

1.3. RNA isolation, reverse transcription and quantitative real-time PCR

For RNA isolation, matrigel containing organoids was dissolved in RA1 buffer and mRNA isolation was performed using the NucleoSpin RNAII kit (Macherey-Nagel, Düren, Germany) according to the manufacturer’s instructions. Reverse transcription and quantitative real-time PCR were performed as previously described [3]. Relative expression of target genes was calculated using the expression of HPRT/Hprt and GAPDH/Gapdh for normalization. Primer sequences are given in Supplementary Table 1.

1.4. Western blot analysis

For protein expression analysis, human and murine organoids were cultivated in hIC and mIC, respectively, for 2-3 days after passaging. Subsequently, medium was changed to CCM for 5 days. Matrigel containing organoids was dissolved in PBS and 5 wells were pooled and centrifuged (300g, 5min, 4°C) to obtain organoid pellets. Supernatants were discarded and organoids were suspended in lysis buffer containing 7M urea, 2M thiourea, 2% CHAPS, 1% DTT (all from Roth, Karlsruhe, Germany) and protease inhibitor (Roche Diagnostics, Mannheim, Germany) and homogenized by ultrasonication. Total protein concentrations were determined using BioRad protein assay (Munich, Germany). Samples were diluted with 5× SDS buffer and 10µg of protein were subjected to electrophoresis on 10% Bis-Tris gels. Proteins were transferred to PVDF membranes (Millipore, Billerica, MA) using a semi dry blotting chamber (Peqlab, Erlangen, Germany), blocked with TBST containing 5% skim milk for 1h and incubated over night at 4°C with antibodies against: PEPT1 (1:3.000, Pineda, custom made) [4], SGLT1 (1:1.000, Pineda, custom made) [3], ChgA (1:1.000, Santa Cruz Biotechnology, Santa Cruz, CA), β-ACTIN (1:1,000; Santa Cruz Biotechnology, Santa Cruz, CA), β-Actin (1:10.000, Cell Signalling Technology, Danvers, MA). Membranes were washed three times and incubated with appropriate IRDye-labeled secondary antibodies (1:10,000; LI-COR Biosciences, Bad Homburg, Germany) for 1.5 h. Proteins were visualized using an infrared scanner (Sapphire Biomolecular Imager, Azure Biosystems) and images were captured with the corresponding Sapphire Capture Software.

1.5. Permeability measurement

Permeability of fluorescence markers into the lumen of organoids was tested using Fluorescein isothiocyanate-dextran (4 kDa) as tracer (Sigma-Aldrich) as described previously [3]. Briefly, matrigel-embedded organoids were incubated in PBS containing FITC-dextran for 30 minutes and subsequently washed with PBS several times before pictures were taken. Permeability tests were performed in human duodenal organoids. Images were taken using a Leica DMI6000 B inverted fluorescence microscope (Leica Microsystems).

1.6. Glucose and Fructose transport studies

For sugar transport studies, human organoids were cultivated for 7-9 days after passaging. Matrigel droplets containing organoids were washed three times with HEPES-saline buffer containing 138mM NaCl, 10mM HEPES, 4.5mM KCl, 4.2mM NaHCO3, 2.6mM CaCl2, 1.2mM NaH2PO4 and 1.2mM MgCl2, pH 7.4. For uptake measurements in intact organoids, organoids still embedded in matrigel were incubated in the before mentioned buffer containing the indicated substrate/inhibitor for 30 min at 37 °C. For uptake measurements of dissociated organoids ("broken up" approach), matrigel droplets containing organoids were washed with PBS, dissolved in GCDR solution (STEMCELL Technologies Grenoble, France) and transferred in tubes. After repeated (2x) centrifugation (200g, 5min) and washing (PBS) steps, dissociated organoids were carefully resuspended and incubated in buffer indicated substrate/inhibitor for 45 min at 37 °C. Transporter activities were assessed using (non-labeled) substrates and 14C-radiolabeled substrate tracers (all American Radiolabeled Chemicals). The following final substrate and inhibitor concentrations were used: 5.8mM D-glucose (final concentration of radiolabeled tracer 2.8mM, specific activity 10mCi/mmol), 2.1mM D-fructose (radiolabeled tracer 100µM, specific activity 300mCi/mmol), 1mM Phloridzin, 1mM Phloretin (both from Sigma) and 1mM Rubusoside (VWR, Avantor). 2.1mM D-glucose was used as competitor in the

fructose uptake assay. Inhibitors were added 10min prior to addition of glucose and 30min prior to addition of fructose. Following incubation, substrate/inhibitor solutions were removed from intact organoids embedded in matrigel and matrigel droplets were washed three times with PBS. Subsequently, matrigel was dissolved by addition of cold PBS and organoids were resuspended and centrifuged (300g, 10min). For "broken up" organoids, dissociated organoids were centrifuged (300g, 3min) and washed three times with PBS. During these steps, PBS for washing contained glucose and fructose, respectively, to avoid excessive transport of substrates out of the cells. Organoid pellets were resuspended in a lysis buffer containing 150mM NaCl, 50 mM Tris-HCl, 1% Igepal CA-630, 0.5% DCA and a protease inhibitor mix, and incubated for 20min on ice. 3 ml of Bioscint® Scintillation solution (National Diagnostics) were added to the cell lysate and radiolabeled substrate taken up by organoids was quantified using a liquid scintillation counter (PerkinElmer). Residual amounts of all radiolabeled tracers were measured in control samples (matrigel alone, without organoids) and were found to be negligible (<1%) compared to the substrates accumulated and measured in the cells of organoids. Glucose uptakes were measured using intact organoids; Fructose uptakes were performed in dissolved organoids derived from the ileum.

1.7. Peptide and drug transport studies

For peptide and drug transport studies, human organoids were cultivated for 7-9 days after passaging. Transport activity was assessed by competitive inhibition of the uptake of radiolabeled dipeptide glycyl-sarcosin (Gly-Sar), a hydrolysis-resistant model substrate of Pept1. The protocol for peptide and drug transport studies resembles the procedure for sugar transport studies with the following modifications: HEPES-saline buffer containing 138mM NaCl, 10mM HEPES, 4.5mM KCl, 4.2mM NaHCO3, 2.6mM CaCl2, 1.2mM NaH2PO4 and 1.2mM MgCl2, had a pH of 6.5. Transporter activities were assessed using (non-labeled) substrates (Gly-Sar) and 14C-radiolabeled substrate tracers (American Radiolabeled Chemicals). Final substrate concentrations used were: 5mM Gly-Sar (radiolabeled tracer 1mM, specific activity 56mCi/mmol), concentration of competitors were: 5mM glycyl-glycine (Gly-Gly) (American Radiolabeled Chemicals), 13mM cyclic hexapeptides, and 20mM Cefadroxil (Sigma). Cyclic hexapeptides were synthetized and provided by Florian Reichart, Michael Weinmüller and Horst Kessler, and solubilized in DMSO. Peptide transporter studies were performed as indicated in murine small intestinal Wt or Pept1-/- organoids or in human duodenal organoids.

1.8. Imaging of intracellular acidification

For live-cell imaging of intracellular acidification, organoids were incubated with 1µM BCECF-AM (Life Technologies) in HEPES-saline buffer containing 0.01% pluronic F127, 375µM eserine and 2mM probenecid for 20 min at 37 °C and then washed 5 times with PBS (pH 7.4; 37 °C). Concentrations of substances used were: 50mM Gly-Sar, 50mM Gly-Gly, 5mM Cefadroxil, 40µM CCCP (Sigma), 100µM Amilorid, and 50µM S1611 (Sanofi Aventis). Image series were captured using a Leica DMI6000 B inverted fluorescence microscope (Leica Microsystems) at 20× magnification every 3–7s and the fluorescence ratio F(λex490nm)/ F(λex450nm) was calculated. Calculations and imaging data analyses were performed using the Leica Application Suite Advanced Fluorescence software. All imaging experiments were performed minimum 6 times and representative time courses are depicted in the manuscript. Imaging of intracellular acidification was performed as indicated in murine small intestinal Wt organoids or in human duodenal organoids.

1.9. *Imaging of intracellular calcium concentrations*

For live-cell imaging of intracellular calcium concentrations, organoids were loaded with 7μM Fura-2 AM (Life Technologies) in HEPES-saline buffer as used for imaging of intracellular acidification for 20 min at 37 °C and then washed 5 times with PBS (pH 7.4; 37 °C). Concentrations of substances used were: 100µM ATP (Sigma), 50mM glucose, and 50mM fructose. Using a Leica DMI6000 B inverted fluorescence microscope (Leica Microsystems) at 20× magnification, images were captured every 5-10s. The fluorescence ratio F(λex340nm)/ F(λex380nm) was calculated and imaging data were analyzed using the Leica Application Suite Advanced Fluorescence software. Imaging of intracellular calcium concentrations was performed in human duodenal organoids.

1.10. *Quantification of amino acid and acylcarnitine concentrations using targeted LC-MS/MS*

Metabolite analyses were performed with samples from three different experiments: 1. Addition of insulin: murine small intestinal organoids were grown for 4 days in CCM and then switched to CCM without N2 supplement (containing insulin) over night. Of note, the B27 supplement contains a n/a concentration of residual insulin. 1µM insulin (final concentration) was added and organoids were incubated for the indicated times. Controls were not pulsed with insulin. 2. Addition of butyrate: murine colonic organoids were grown for 6 days in IC. 100µM carnitine and 1mM butyrate were added and organoids were incubated for the indicated times. Time point zero was sampled immediately after addition of butyrate and carnitine. Control organoids were not exposed to butyrate. 3. Addition of d13-labeled palmitate: murine small intestinal organoids were grown for 4 days in CCM and then switched to CCM prepared with low glucose DMEM/F12 medium (Gibbco, Cincinnati, OH) for 24h. 200µM carnitine, 0.3% BSA and 100µM d31-labeled palmitate (final concentrations) were added and organoids were incubated for the indicated times. Controls were exposed to carnitine and BSA without palmitate. Medium from untreated organoids was measured to verify organoids as source of metabolites.

For metabolite analysis, organoids were washed, resuspended in cold PBS and centrifuged (300g, 5min) to remove remaining matrigel. 500µL ice-cold methanol containing a mixture of 13 deuterium-labeled amino acid and acylcarnitine species (standard) was added to the organoid pellet. Samples were centrifuged (10,000g, 10min, 4°C) and 400µL of the supernatant was collected and dried in nitrogen gas. Amino acids and acylcarnitines were derivatized to their butyl esters as described elsewhere [5]. Briefly, a mixture of 95% n-butanol and 5% acetylchloride (v/v) was added to the samples. Samples were subsequently incubated at 60°C for 15min while shaken at 600rpm (Eppendorf Thermomixer Comfort; Eppendorf, Hamburg, Germany). The samples were dried in nitrogen gas and reconstituted in a 300µl mixture of methanol/water/formic acid (70/30/0.1% v/v). To determine amino acid and acylcarnitine concentrations, quantitative LC-MS/MS was applied as described previously [6, 7]. A triple quadrupole QTRAP 5500 LC-MS/MS system (Sciex, Framingham, MA, USA) equipped with a 1200 series binary pump (Agilent, Santa Clara, CA) and coupled to an HTC pal autosampler (CTC Analytics, Zwingen, Switzerland) was used. Data analysis was performed using Analyst 1.7® and SCIEX OS 1.4 software (Sciex).

1.11. Proteasome activity measurement

Measurement of proteasome activity was performed using the Proteasome-Glo Cell-Based Assay (Chymotrypsin-Like Assay), and Life-cell protease activity was measured using the CellTiter-Glo Luminescent Cell Viability Assay (both from Promega, Mannheim, Germany), according to the manufacturer's instructions. Fluorescence and luminescence were measured using the Tecan infinite M200 (Tecan Group Ltd., Männedorf, Switzerland) and the i-control Microplate Reader software (Tecan, Grödig, Austria).

1.12. Statistical analysis

Statistical computations were performed using GraphPad Prism (GraphPad, La Jolla, CA). Statistical tests used were: unpaired t-test for two group-comparisons or One-Way analysis of variances followed by an appropriate multiple comparison procedure for comparisons comprising more than two groups. Data are expressed as mean ± standard error mean, $n \geq 5$ for all groups unless otherwise stated. Differences between groups were considered significant if P-values were < 0.05. P values are either given or (*) indicates P-values < 0.05, (**) indicates P-values < 0.01, and (***) indicates P-values < 0.001.

2. Supplementary Table 1: *Primer sequences for quantitative real-time PCR*

Human Gene	Primer Sequences	Probe #
ALPI	CATGGACCGCTTCCCATA<br>GGCACCTGTCTGTCCACAT	21
CHGA	CAAACCGCAGACCAGAGG<br>TCCAGCTCTGCTTCAATGG	17
GAPDH	AGCCACATCGCTCAGACAC<br>GCCCAATACGACCAAATCC	60
GATA4	GGAAGCCCAAGAACCTGAAT<br>GTTGCTGGAGTTGCTGGAA	69
GCG (GLP-1)	GTACAAGGCAGCTGGCAAC<br>TGGGAAGCTGAGAATGATCTG	82
HPRT	TGATAGATCCATTCCTATGACTGTAGA<br>CAAGACATTCTTTCCAGTTAAAGTTG	22
SCNN1A (ENaC)	TGTGACTACAGAAAGCACAGTTCC<br>CCAGGTGGTCTGAGGAGAAGT	1
SLC2A2 (GLUT2)	CCCTGTCTGTATCCAGCTTTG<br>TGTTTGCTACTAACATGGCTTTG	31
SLC2A5 (GLUT5)	TCTCCTTGCAAACGTAGATGG<br>GAAGAAGGGCAGCAGAAGG	3
SLC5A1 (SGLT1)	TGGCAATCACTGCCCTTTA<br>TGCAAGGTGTCCGTGTAAAT	70
SLC9A3 (NHE3)	CTCCGAAGCTGGCAAGAA<br>CATAGCCCTGAGGTCCCTTT	24
SLC10A2 (ABST)	TATAGGATGCTGCCCTGGAG<br>GCAGTGTGGAGCATGTGG	16
SLC15A1 (PEPT1)	CAATGTTCTGGGCCTTGTTT<br>CCGATTTTCCCGGACATAG	1
SLC51B (OSTB)	AGCATCCAGGCAAGCAGA<br>CCTCATCCAAATGCAGGACT	83
SPINK1	GGGACTTTTCTCCCTTTTGC<br>CTCCAGGTTCTGGGAATGTC	74

Murine Gene	Primer Sequences	Probe #
Alpi	catctccaacatggacattga<br>ggttccagactggttactgtca	109
Chga	ggaggctgtggctagagaga<br>ccatccactgcctgagagtc	89
Gapdh	tccactcatggcaaattcaa<br>tttgatgttagtggggtctcg	9
Gcg (Glp-1)	ccagtgatgtgagttcttacttgg<br>caatggcgacttcttctgg	27
Hprt	tcctcctcagaccgctttt<br>cctggttcatcatcgctaatc	95
Slc5a1 (Sglt1)	ctggcaggccgaagtatg<br>ttccaatgttactggcaaagag	49
Slc15a1 (Pept1)	agctctgatcgcagactcgt<br>cgtgtagacgatggatagtgaaa	60

3. Supplementary Figures

3.1. Supplementary Figure 1

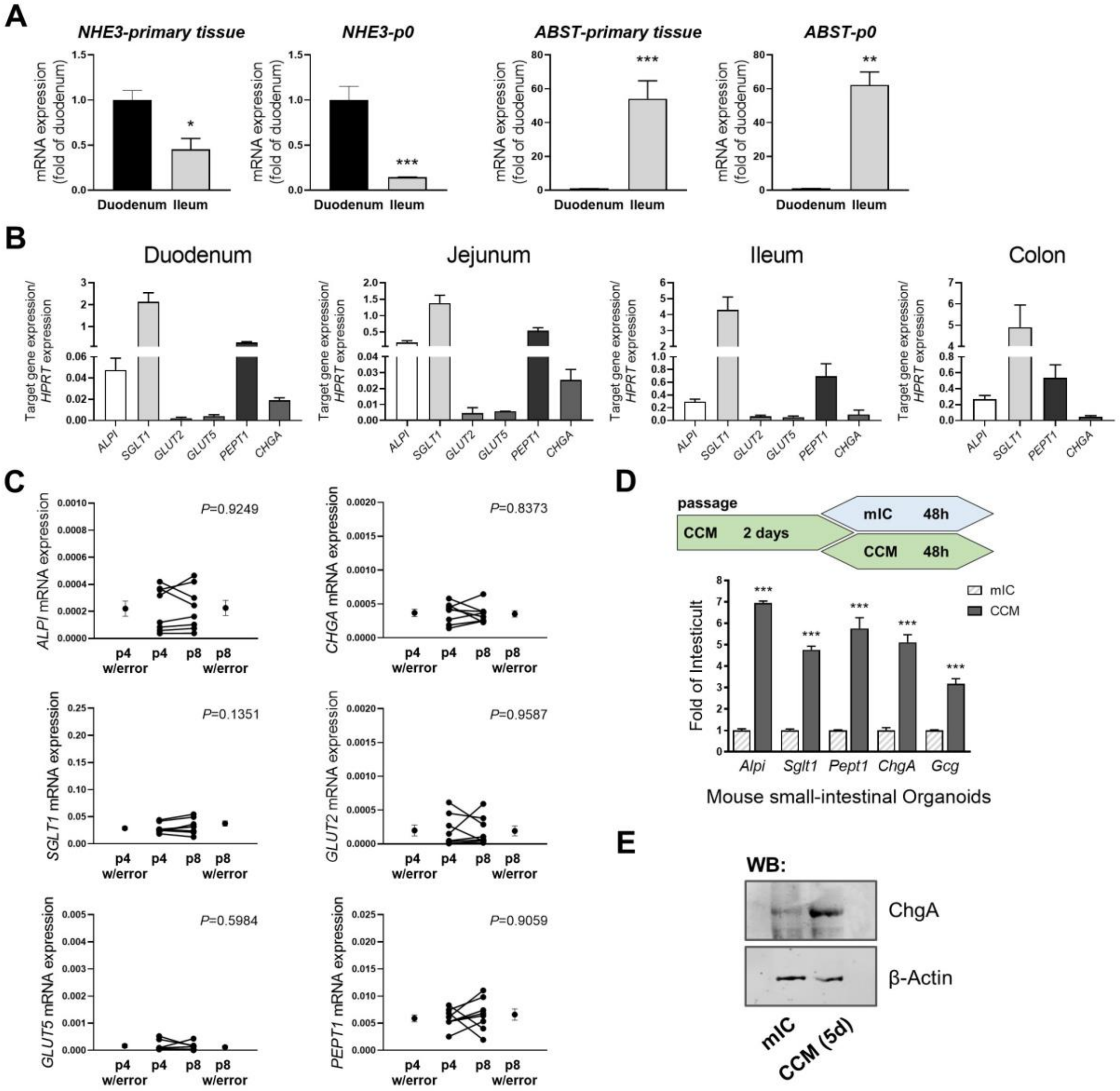

Supplementary Figure 1 Culture conditions impact intestinal organoid cell composition and expression of nutrient transporters. (**A,B**) mRNA expression analyses of primary tissue (**A**) and human organoids (**A,B**) derived from different intestinal segments; IEC-transport and subtype-related genes (*ALPI*= enrerocyte; *CHGA*= enterodendocrine cells) are depict (passage p0). (**A**) NHE3-p0 and ABST-p0 correspond to Fig.1 and are shown for comparison. (**C**) mRNA expression levels of duodenal human organoids from passages p4 and p8. (**A-C**) Target gene expression normalized to *HPRT*. (**D**) Upper panel: Schematic representation of the experimental setup from which samples were derived for subsequent mRNA expression analysis. Lower panel: Relative gene expression of CCM-cultured murine small intestinal organoids as fold of organoids cultured in Wnt-containing mIC medium. *Hprt* was used as housekeeper. Bars represent mean +SEM. (**E**) Protein expression of ChgA in murine organoids cultured in murine Intesticult medium (mIC) and crypt culture medium (CCM) medium for 5 days, respectively. β-Actin serves as loading control. (**A,D**) unpaired t tests (n=6). (**C**) paired t tests (n=5-6). Asterisks indicate significant differences *P<0.05, **P<0.01, ***P<0.001.

3.2. *Supplementary Figure 2*

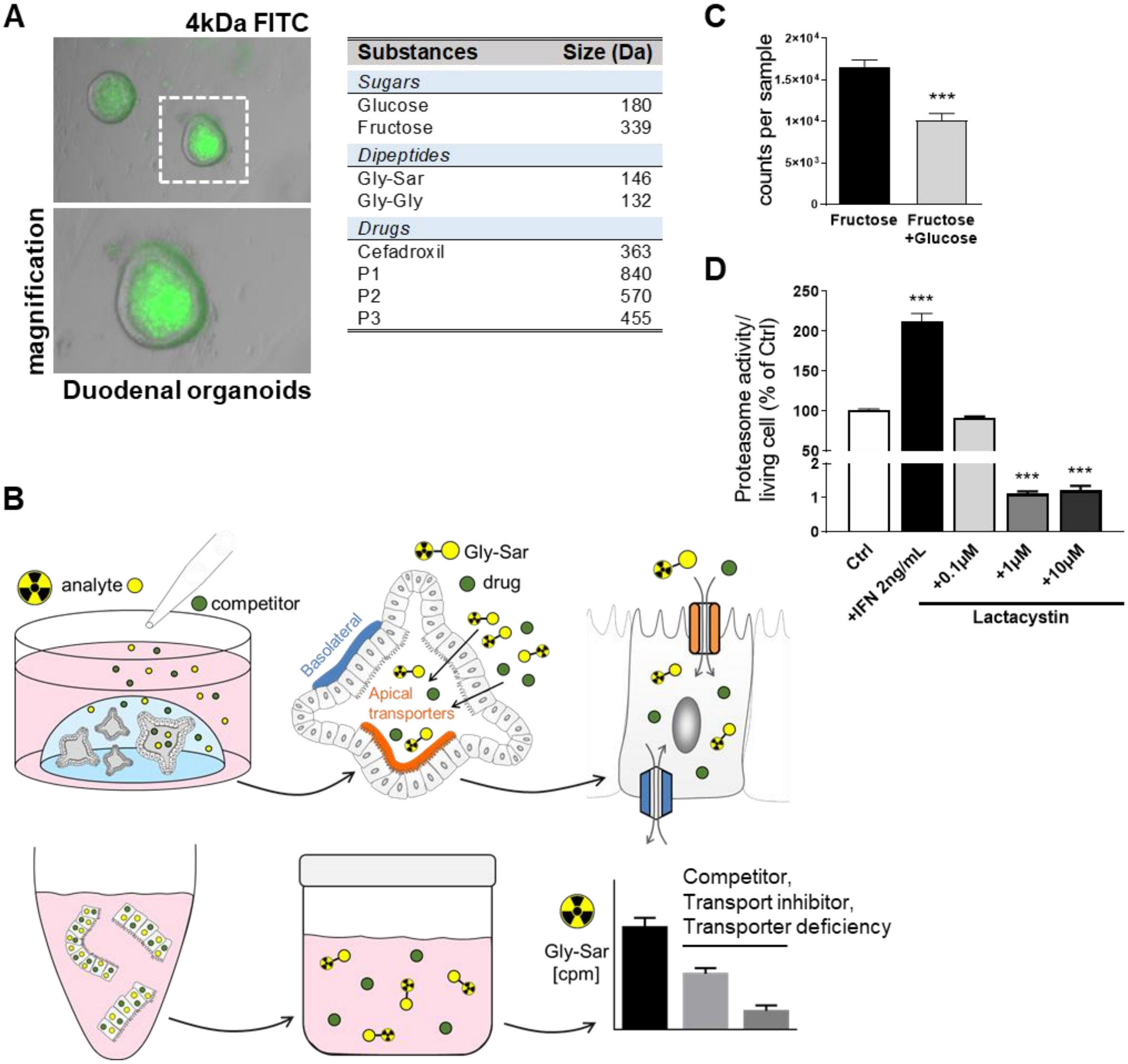

Substances	Size (Da)
Sugars	
Glucose	180
Fructose	339
Dipeptides	
Gly-Sar	146
Gly-Gly	132
Drugs	
Cefadroxil	363
P1	840
P2	570
P3	455

Supplementary Figure 2 Nutrient and drug transport in human intestinal organoids. (**A**) Representative pictures showing luminal FITC-Dextran (4 kDa) in human duodenal organoids after addition of FITC-Dextran to the growth medium (basolateral side) (**B**) Schematic representation of the experimental procedures for measuring transport activity using radiolabeled substrates („intact organoids" approach). (**C**) Inhibition of fructose uptake using glucose as competitor in human duodenal organoids. (**D**) Proteasome activity normalized to life cell protease activity in murine small intestinal organoids. Interferon γ was used as positive control, lactacystin as specific inhibitor of proteasome activity. Bars represent mean +SEM. (**C**) Unpaired t test (n=5-6). (**D**) One-way analysis of variance (ANOVA) followed by Tukey's test (n=5). Asterisks indicate significant differences compared to control, ***P<0.001.

3.3. Supplementary Figure 3

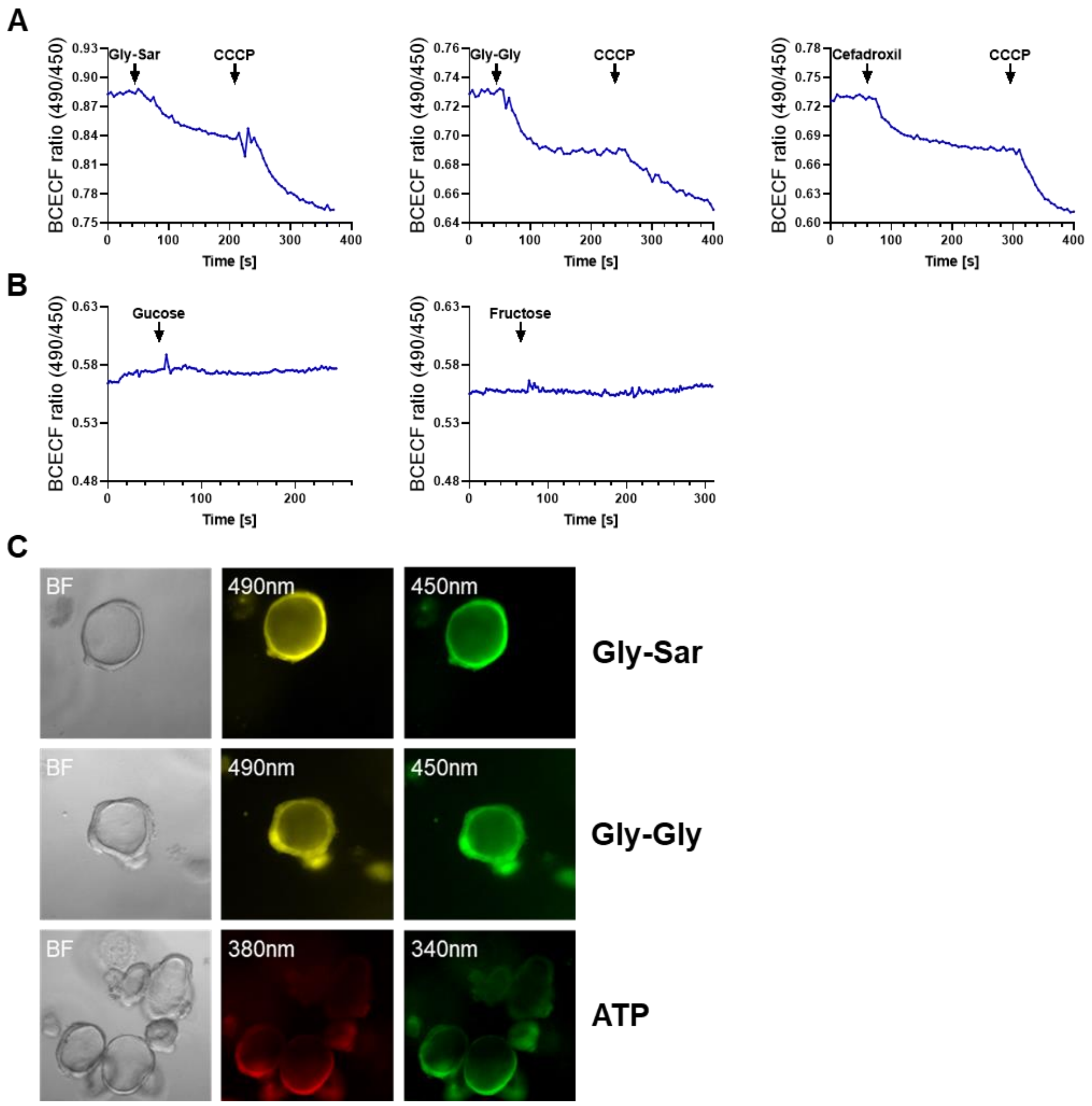

Supplemental Figure 3 Visualization of intestinal peptide transport processes. (**C**) Pictures of organoids corresponding to the measurements shown in figure 3A, B, and D. BF: bright-field; 490/450 nm (excitation wavelengths of BCECF-AM); 340/380 nm (excitation wavelengths of Fura-2). (**B**) Negative control to measurements depict in figure 3. Addition of glucose or fructose did not change intracellular proton levels as visualized by BCECF-AM measurements. (**A**) Intracellular acidification visualized by BCECF-AM induced by transport of peptide-transporter substrates (left) Gly-Sar, (middle) Gly-Gly or (right) the antibiotic Cefadroxil and subsequent forced intracellular acidification by the protonophore CCCP, demonstrating a physiological range of responses to Gly-Sar, Gly-Gly, and Cefadroxil. (**A,B**) For data analysis, whole organoids were selected and no background correction was applied. Analyses were performed on several organoids derived from independent cultures and representative measurements are shown.

4. References

1. Zietek, T. and E. Rath, *Chapter 3 - Intestinal organoids: Mini-guts grown in the laboratory*, in *Organs and Organoids*, J.A. Davies and M.L. Lawrence, Editors. 2018, Academic Press. p. 43-71.
2. Hu, Y., et al., *Targeted disruption of peptide transporter Pept1 gene in mice significantly reduces dipeptide absorption in intestine.* Mol Pharm, 2008. **5**(6): p. 1122-30.
3. Zietek, T., et al., *Intestinal organoids for assessing nutrient transport, sensing and incretin secretion.* Sci Rep, 2015. **5**: p. 16831.
4. Wuensch, T., et al., *Colonic expression of the peptide transporter PEPT1 is downregulated during intestinal inflammation and is not required for NOD2-dependent immune activation.* Inflamm Bowel Dis, 2014. **20**(4): p. 671-84.
5. Gucciardi, A., et al., *A rapid UPLC-MS/MS method for simultaneous separation of 48 acylcarnitines in dried blood spots and plasma useful as a second-tier test for expanded newborn screening.* Anal Bioanal Chem, 2012. **404**(3): p. 741-51.
6. Giesbertz, P., et al., *An LC-MS/MS method to quantify acylcarnitine species including isomeric and odd-numbered forms in plasma and tissues.* J Lipid Res, 2015. **56**(10): p. 2029-39.
7. Harder, U., B. Koletzko, and W. Peissner, *Quantification of 22 plasma amino acids combining derivatization and ion-pair LC-MS/MS.* J Chromatogr B Analyt Technol Biomed Life Sci, 2011. **879**(7-8): p. 495-504.
